# Survival benefit and impact of adjuvant chemotherapy following systemic neoadjuvant chemotherapy in patients with resected pancreas ductal adenocarcinoma: a retrospective cohort study

**DOI:** 10.1097/JS9.0000000000000589

**Published:** 2023-07-06

**Authors:** Ning Pu, Wenchuan Wu, Siyao Liu, Yuqi Xie, Hanlin Yin, Qiangda Chen, Taochen He, Zhihang Xu, Wenquan Wang, Jun Yu, Liang Liu, Wenhui Lou

**Affiliations:** a Department of Pancreatic Surgery; b Cancer Center; c Department of General Surgery, Zhongshan Hospital, Fudan University, Shanghai, People’s Republic of China; d Departments of Medicine and Oncology, Johns Hopkins University School of Medicine, Baltimore, Maryland, USA

**Keywords:** adjuvant chemotherapy, neoadjuvant chemotherapy, pancreatic ductal adenocarcinoma, prognosis

## Abstract

**Background:**

Patients with pancreatic ductal adenocarcinoma (PDAC) are increasingly receiving systemic neoadjuvant chemotherapy (NAC), particularly those with borderline resectable and locally advanced disease. However, the specific role of additional adjuvant chemotherapy (AC) in these patients is unknown. The objective of this study is to further assess the clinical benefit and impact of systemic AC in patients with resected PDAC after NAC.

**Methods:**

Data on PDAC patients with or without AC following systemic NAC and surgical resection were retrospectively retrieved from the Surveillance, Epidemiology, and End Results (SEER) database between 2006 and 2019. A matched cohort was created using propensity score matching (PSM), and baseline characteristics were balanced to reduce bias. Overall survival (OS) and cancer-specific survival (CSS) were calculated using matching cohorts.

**Results:**

The study enrolled a total of 1589 patients, with 623 (39.2%) in the AC group and 966 (51.8%) in the non-AC group [mean age, 64.0 (9.9) years; 766 (48.2%) were females and 823 (51.8%) were males]. All patients received NAC, and among the crude population, 582 (36.6%) received neoadjuvant radiotherapy, while 168 (10.6%) received adjuvant radiotherapy. Following the 1:1 PSM, 597 patients from each group were evaluated further. The AC and non-AC groups had significantly different median OS (30.0 vs. 25.0 months, *P*=0.002) and CSS (33.0 vs. 27.0 months, *P*=0.004). After multivariate Cox regression analysis, systemic AC was independently associated with improved survival (*P*=0.003, HR=0.782; 95% CI, 0.667–0.917 for OS; *P*=0.004, HR=0.784; 95% CI, 0.663–0.926 for CSS), and age, tumor grade, and AJCC N staging were also independent predictors of survival. Only patients younger than 65 years old and those with a pathological N1 category showed a significant association between systemic AC and improved survival in the subgroup analysis adjusted for these covariates.

**Conclusion:**

Systemic AC provides a significant survival benefit in patients with resected PDAC following NAC compared to non-AC patients. Our study discovered that younger patients, patients with aggressive tumors and potentially well response to NAC might benefit from AC to achieve prolonged survival after curative tumor resection.

## Introduction

Highlights

- Adjuvant chemotherapy after neoadjuvant chemotherapy (NAC) and surgical resection was associated with survival benefits in pancreatic ductal adenocarcinoma (PDAC).
- Younger PDAC patients may be benefited from adjuvant chemotherapy after NAC and surgery.
- Patients with pathological N1 category after NAC showed significant association between adjuvant therapy and improved survival.

Pancreatic ductal adenocarcinoma (PDAC) is a highly aggressive and lethal disease with a 5-year overall survival (OS) rate of less than 12%^1^. Due to its high cancer-related mortality and increasing incidence, PDAC is expected to become the second leading cause of death worldwide by 2030^2^. For several decades, PDAC has been widely assumed to be curable only through surgical resection^3^. However, even with successful resection, the 5-year OS rate for patients is as low as 20%, and the median OS is only 24.1 months, which is significantly lower than the desired outcome^4,5^. Adjuvant chemotherapy (AC) with gemcitabine, oxaliplatin, irinotecan, fluorouracil, and leucovorin (FOLFIRINOX) is currently being studied for PDAC, and reveals some efficacy^6–10^.

In recent years, neoadjuvant therapy (NAT) has become an important treatment option for localized cancer, including borderline resectable and locally advanced PDAC^11,12^. Recent pooled analyses have shown that when compared to upfront surgery followed by AC, neoadjuvant chemotherapy (NAC) does not improve disease-free survival (DFS) or OS in patients with resectable PDAC. NAC has been reported to increase R0 rates by 20%^13,14^. The NEONAX trial found that patients with resectable PDAC treated with NAC and AC had a longer OS than those treated with AC alone, suggesting that neoadjuvant/perioperative treatment may be a viable alternative to AC for patients with resectable PDAC^15^. However, the precise role of AC following NAC remains unclear. AC after neoadjuvant FOLFIRINOX and surgical resection, according to van Roessel *et al*.^16^, was associated with improved survival only in patients with positive lymph node metastases in their large, multicenter, retrospective study. The benefits of AC in patients treated with multi-agent NAC and surgical resection were further validated using propensity score matching (PSM) from the National Cancer Database (NCDB) data collected by Sugawara *et al*.^17^. In PDAC patients, AC after multi-agent NAC and resection was correlated with significant survival benefits compared to patients who did not receive AC after multi-agent NAC and resection.

Since most retrospective studies on the benefits of AC in patients with resected PDAC used data from NCDB and not randomized clinical trials (RCTs), it would be helpful to further validate these findings with a large, retrospective study from another extensive, authoritative database. As a result, the goal of this study is to further validate the findings of van Roessel *et al*.^16^ and Sugawara *et al*.^17^ and evaluate the use of systemic AC in patients with resected PDAC after systemic NAC, using a propensity score-matched data set obtained from the Surveillance, Epidemiology, and End Results (SEER) database.

## Methods

### Data source and patient population

All patients in this study were obtained from the National Cancer Institute’s SEER database registry (https://seer.cancer.gov/). The SEER*Stat Database: Incidence – SEER Research Plus Data, 17 Registries, Nov 2021 Sub (2020–2019) was used as the most recent database. After access was granted, all data were freely available for research in the SEER database. The study was conducted in compliance with the 1964 Helsinki Declaration and reported in accordance with the STROCSS criteria (Supplemental Digital Content 7, http://links.lww.com/JS9/A756), which was registered in Prospero^18^. According to the Ethics Committee of our institute, this study was not considered human participant research and did not require institutional review board approval because it used de-identified data from secondary research.

**Figure 1:**
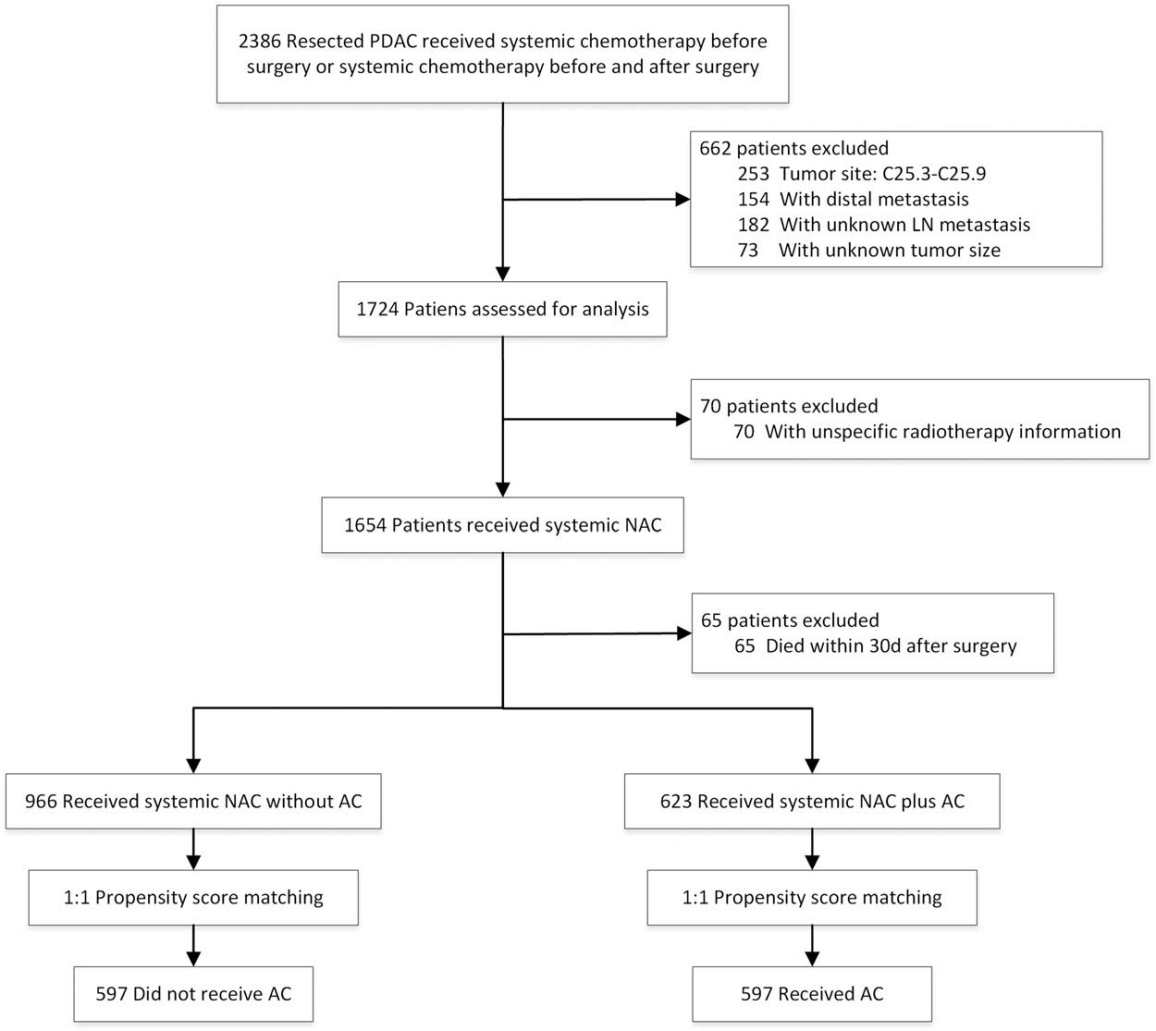
Flowchart of study cohort.

A subset of resected PDAC patients who received systemic chemotherapy either before surgery or both before and after surgery were enrolled. Patients with C25.3–C25.9, clinical or pathological stage IV disease, missing lymph node metastasis or tumor size information, or unspecific or inconsistent radiotherapy information were excluded. We also excluded patients with a survival time of less than one month because they were more likely to die or be censored due to perioperative complications (Fig. 1). Following the inclusion and exclusion criteria, the crude data set comprised the entire enrolled cohort.

For each patient, the following information was gathered: Age, Sex, Year of diagnosis, Race, Primary site, Grade, RX Summ--Surg/Rad Seq, Chemotherapy recode (yes, no/unk), RX Summ--Systemic/Sur Seq, Derived EOD 2018 T (2018+), Derived EOD 2018 N (2018+), Derived EOD 2018 M (2018+), Derived EOD 2018 Stage Group (2018+), Derived AJCC T, 7th ed (2010-2015), Derived AJCC N, 7th ed (2010-2015), Derived AJCC M, 7th ed (2010-2015), Derived AJCC Stage Group, 7th ed (2010-2015), Derived SEER Combined T (2016-2017), Derived SEER Combined N (2016-2017), Derived SEER Combined M (2016-2017), Derived SEER Cmb Stg Grp (2016-2017), Derived AJCC T, 6th ed (2004-2015), Derived AJCC N, 6th ed (2004-2015), CS mets at dx (2004-2015), Derived AJCC M, 6th ed (2004-2015), Derived AJCC Stage Group, 6th ed (2004-2015), EOD Primary Tumor (2018+), EOD Regional Nodes (2018+), EOD Mets (2018+), Tumor Size Summary (2016+), CS tumor size (2004-2015), CS lymph nodes (2004-2015), CS extension (2004-2015), SEER Combined Mets at DX-bone (2010+), SEER Combined Mets at DX-brain (2010+), SEER Combined Mets at DX-liver (2010+), SEER Combined Mets at DX-lung (2010+), Mets at DX-Distant LN (2016+), Mets at DX-Other (2016+), Regional nodes examined (1988+), Regional nodes positive (1988+), Marital status at diagnosis, and survival information. These above codes were used to evaluate the 8th edition of the AJCC TNM stage.

## Statistical analysis

All statistical analyses in this study were carried out using SPSS 21.0 software (IBM Corporation, Armonk, New York, USA) and R software version 4.0.5 (http://www.r-project.org/). The *χ* ^2^ test or Fisher’s exact test was used to examine the relationships between AC and categorical variables. Next, the PSM method was then used, and the AC group was marched to the non-AC group using the MatchIt package in the R project, with a 1:1 nearest-neighbor PSM algorithm that took all potential confounders into account (caliper width =0.1SD). Radiotherapy, as previously reported, was not included in our correlation analysis^17^. Furthermore, the regimen types were not identified in the model due to a lack of precise data on systemic NAC and AC in the SEER database. However, we included period and radiotherapy as covariates in the Cox proportional hazards models, and time was divided into three periods, as previously reported: 2006–2011, 2012–2014, and 2015–2019^17^.

Kaplan–Meier curves with log-rank tests were used to analyze unadjusted OS and cancer-specific survival (CSS) using GraphPad Prism 8 Software (GraphPad Software Inc., San Diego, California, USA). OS was defined as the time between surgery and the date of death or last follow-up, whereas CSS was defined as the time between surgery and the date of cancer-related death or last contact. The independent prognostic indicators were identified using univariate and multivariate Cox proportional hazards regression models. Furthermore, we examined the interactions between AC and each significant prognostic variable using a single multivariable Cox regression model that was adjusted for all factors. For each interaction coefficient in the non-AC group, hazard ratios (HRs) and 95% confidence intervals (CIs) were calculated. All *P* values less than 0.05 were considered statistically significant.

## Results

### Demographic and clinicopathological characteristics

This study analyzed 1589 histologically confirmed PDAC patients [mean (SD) age, 64.0 (9.9) years; 766 (48.2%) female and 823 (51.8%) male] who met the inclusion and exclusion criteria (Fig. 1). All enrolled patients received NAC and/or AC. Of the patients, 582 (36.6%) received neoadjuvant radiotherapy, and 168 (10.6%) received adjuvant radiotherapy. Six hundred twenty-three patients (39.2%) received AC (AC group), while 966 (60.8%) did not (non-AC group). The patients were classified as having stage IA (96), IB (441), IIA (144), IIB (449), and III (459) disease, respectively. After conducting a correlation analysis, it was discovered that AJCC T staging (*P*=0.002), AJCC N staging (*P*<0.001), and AJCC 8th TNM staging system (*P*<0.001) were significantly associated with AC (Table 1).

**Table 1:** Demographic and clinicopathological characteristics of cohorts with or without systematic adjuvant chemotherapy after neoadjuvant chemotherapy and surgery

	Groups, *N* (%)					
	Crude data set			Matched data set		
Variables	Non-AC (*n*=966)	AC (*n*=623)	*P*	Non-AC (*n*=597)	AC (*n*=597)	*P*
Age			0.131			0.381
≤65	510 (52.8)	353 (56.7)		332 (55.6)	347 (58.1)	
>65	456 (47.2)	270 (43.3)		265 (44.4)	250 (41.9)	
Sex			0.504			0.487
Female	459 (47.5)	307 (49.3)		282 (47.2)	294 (49.2)	
Male	507 (52.5)	316 (50.7)		315 (52.8)	303 (50.8)	
Grade			0.076			0.052
Well	43 (4.5)	28 (4.5)		29 (4.9)	26 (4.4)	
Moderate	162 (16.8)	137 (22.0)		97 (16.2)	132 (22.1)	
Poor/undifferentiated	137 (14.2)	81 (13.0)		92 (15.4)	74 (12.4)	
Unknown	624 (64.6)	377 (60.5)		379 (63.5)	365 (61.1)	
Race			0.447			0.543
Black	98 (10.1)	55 (8.8)		60 (10.1)	51 (8.5)	
White	807 (83.5)	519 (83.3)		498 (83.4)	498 (83.4)	
Asian or Pacific Islander	53 (5.5)	45 (7.2)		34 (5.7)	44 (7.4)	
Others	8 (0.8)	4 (0.6)		5 (0.8)	4 (0.7)	
Tumor site			0.513			0.523
Head	830 (85.9)	532 (85.4)		521 (87.3)	508 (85.1)	
Body	92 (9.5)	55 (8.8)		48 (8.0)	54 (9.0)	
Tail	44 (4.6)	36 (5.8)		28 (4.7)	35 (5.9)	
T classification			**0.002**			0.582
T1	102 (10.6)	64 (10.3)		74 (12.4)	59 (9.9)	
T2	515 (53.3)	385 (61.8)		354 (59.3)	364 (61.0)	
T3	186 (19.3)	104 (16.7)		99 (16.6)	104 (17.4)	
T4	163 (16.9)	70 (11.2)		70 (11.7)	70 (11.7)	
N classification			**<0.001**			1.000
N0	575 (59.5)	244 (39.2)		244 (40.9)	244 (40.9)	
N1	271 (28.1)	251 (40.3)		246 (41.2)	246 (41.2)	
N2	120 (12.4)	128 (20.5)		107 (17.9)	107 (17.9)	
TNM staging system			**<0.001**			0.999
IA	61 (6.3)	35 (5.6)		33 (5.5)	35 (5.9)	
IB	302 (31.3)	139 (22.3)		141 (23.6)	139 (23.3)	
IIA	104 (10.8)	40 (6.4)		40 (6.7)	40 (6.7)	
IIB	230 (23.8)	219 (35.2)		215 (36.0)	214 (35.8)	
III	269 (27.8)	190 (30.5)		168 (28.1)	169 (28.3)	
Marital status			0.501			0.440
Married	634 (65.6)	425 (68.2)		397 (66.5)	413 (69.2)	
Single (never married)	117 (12.1)	74 (11.9)		66 (11.1)	68 (11.4)	
Others	215 (22.3)	124 (19.9)		134 (22.4)	116 (19.4)	

Statistical significance *P* values in bold.

A PSM model was used to reduce confounders and reflect the nature of the two strategies. The distribution of all included variables was adequately balanced after 1:1 PSM adjusted by all potential confounders (Table 1). Finally, 597 non-AC patients and 597 AC patients were perfectly matched. Figure S1 (Supplemental Digital Content 1, http://links.lww.com/JS9/A750) shows the distribution and histograms of propensity scores before and after matching.

### Relationship between systemic adjuvant chemotherapy with survival in the matched data

**Figure 2:**
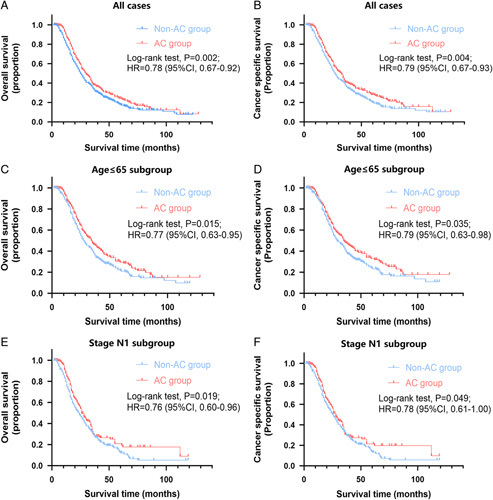
Overall survival and cancer-specific survival stratified by adjuvant chemotherapy (AC) in all matched patients (A and B), age ≤65 subgroup patients (C and D), and stage N1 subgroup patients (E and F).

The median OS and CSS of the matched population were 27.0 (IQR: 16.0–52.0) and 29.0 (IQR: 17.0–58.0) months, respectively. The 1-year, 3-year, and 5-year OS rates were 85.7%, 36.3%, and 20.6%, respectively, while the CSS rates were 87.1%, 39.4%, and 23.6%. Further investigation revealed that the median OS of the AC group was significantly greater than that of the non-AC group [30.0 (IQR: 18.0–58.0) vs. 25.0 (IQR: 15.0–48.0) months, *P*=0.002; Fig. 2A]. The OS rates at 1, 3, and 5 years in the AC group were 90.2%, 38.2%, and 24.1%, respectively, while in the non-AC group, they were 81.1%, 34.2%, and 17.5%, respectively. With regard to CSS, the median CSS of the AC group was also significantly longer [33.0 (IQR: 19.0–67.0) vs. 27.0 (IQR: 16.0–53.0) months, *P*=0.004; Fig. 2B]. The cumulative CSS rates in the AC group at 1, 3, and 5 years were 90.6%, 40.9%, and 28.0%, respectively, whereas they were 83.5%, 37.7%, and 19.8% in the non-AC group.

In the matched data, older age, higher grade, advanced T stage, advanced N stage, advanced TNM stage, and systemic non-AC were all identified as risk factors for both OS and CSS. Systemic AC was significantly associated with better survival after multivariate analysis (*P*=0.003, HR=0.782; 95% CI, 0.667–0.917 for OS; *P*=0.004, HR=0.784; 95% CI, 0.663–0.926 for CSS) compared to those who did not receive AC. In addition, higher mortality rates were independently correlated with being over the age of 65, having a poor/undifferentiated tumor, and having a pathological N category of N1 or higher (Table 2).

**Table 2:** Univariate and multivariate Cox regression analysis for overall survival and cancer-specific survival in the matched data

	Overall survival			Cancer-specific survival		
Variables	Univariate *P*	Multivariate *P*	Hazard ratio (95% CI)	Univariate *P*	Multivariate *P*	Hazard ratio (95% CI)
Age						
≤65	Ref.	Ref.	Ref.	Ref.	Ref.	Ref.
>65	**<0.001**	**<0.001**	1.525 (1.298–1.792)	**<0.001**	**<0.001**	1.489 (1.257–1.764)
Sex						
Female	Ref.			Ref.		
Male	0.078			0.087		
Grade						
Well	Ref.	Ref.	Ref.	Ref.	Ref.	Ref.
Moderate	0.188	0.136	1.322 (0.916–1.908)	0.213	0.174	1.305 (0.889–1.916)
Poor/undifferentiated	**0.001**	**0.002**	1.829 (1.259–2.658)	**0.003**	**0.004**	1.770 (1.198–2.617)
Unknown	0.743	0.657	1.084 (0.760–1.546)	0.792	0.727	1.068 (0.737–1.549)
Race						
Black	Ref.			Ref.		
White	0.090			0.069		
Asian or Pacific Islander	0.530			0.514		
Others	0.457			0.576		
Tumor site						
Head	Ref.			Ref.		
Body	0.782			0.556		
Tail	0.282			0.518		
Year of diagnosis						
2006–2011	Ref.			Ref.		
2012–2014	0.229			0.208		
2015–2019	0.347			0.161		
Neoadjuvant radiotherapy						
No	Ref.			Ref.		
Yes	0.957			0.997		
Adjuvant radiotherapy						
No	Ref.			Ref.		
Yes	0.757			0.416		
T classification						
T1	Ref.	Ref.	Ref.	Ref.	Ref.	Ref.
T2	**0.008**	0.264	1.216 (0.863–1.715)	**0.003**	0.078	1.403 (0.962–2.045)
T3	**0.007**	0.190	1.307 (0.876–1.951)	**0.002**	0.051	1.537 (0.998–2.367)
T4	0.150	0.243	1.491 (0.763–2.914)	**0.043**	0.072	1.883 (0.945–3.751)
N classification						
N0	Ref.	Ref.	Ref.	Ref.	Ref.	Ref.
N1	**<0.001**	**0.050**	1.662 (1.001–2.760)	**<0.001**	**0.041**	1.731 (1.023–2.930)
N2	**<0.001**	**0.003**	2.886 (1.441–5.780)	**<0.001**	**0.001**	3.161 (1.561–6.398)
TNM staging system						
IA	Ref.	Ref.	Ref.	Ref.	Ref.	Ref.
IB	**0.048**	0.253	1.384 (0.793–2.416)	0.072	0.581	1.185 (0.649–2.162)
IIA	**0.022**	0.397	1.317 (0.696–2.492)	**0.034**	0.751	1.117 (0.563–2.215)
IIB	**<0.001**	0.645	1.185 (0.575–2.446)	**<0.001**	0.861	1.071 (0.497–2.309)
III	**<0.001**	0.806	0.896 (0.372–2.156)	**<0.001**	0.524	0.743 (0.298–1.853)
Marital status						
Married	Ref.			Ref.		
Single (never married)	0.333			0.816		
Others	0.652			0.560		
Adjuvant chemotherapy						
No	Ref.	Ref.	Ref.	Ref.	Ref.	Ref.
Yes	**0.002**	**0.003**	0.782 (0.667–0.917)	**0.004**	**0.004**	0.784 (0.663–0.926)

Statistical significance *P* values in bold.

### Subgroup interaction analysis

The interaction analysis was carried out to identify subgroups that might benefit differently from systemic AC. In the unadjusted subgroup analysis of age, we found that the association with systemic AC was significant only in patients younger than 65 years old (*P*=0.015, HR=0.77; 95% CI, 0.63–0.95 for OS; *P*=0.035, HR=0.79; 95% CI, 0.63–0.98 for CSS; Fig. 2C, D and Fig. S2, Supplemental Digital Content 2, http://links.lww.com/JS9/A751). When analyzing the unadjusted subgroups of pathological characteristics, we discovered that patients with grade 3 tumors were associated with AC referring to total mortality (*P*=0.038, HR=0.71; 95% CI, 0.51–0.99; Fig. S3E, Supplemental Digital Content 3, http://links.lww.com/JS9/A752) rather than cancer-specific mortality (*P*=0.069, HR=0.73; 95% CI, 0.52–1.03; Fig. S3F, Supplemental Digital Content 3, http://links.lww.com/JS9/A752). Those with a pathological N1 category were significantly associated with AC (*P*=0.019, HR=0.76; 95% CI, 0.60–0.96 for OS; *P*=0.049, HR=0.78; 95% CI, 0.61–1.00 for CSS; Fig. 2E, F), and those with a pathological T2 category was also significantly associated with AC (*P*=0.018, HR=0.79; 95% CI, 0.64–0.96 for OS; *P*=0.022, HR=0.78; 95% CI, 0.63–0.97 for CSS; Fig. S5C, D, Supplemental Digital Content 4, http://links.lww.com/JS9/A753). However, patients with grade 1 or 2 tumors (Fig. S3A–D, Supplemental Digital Content 3, http://links.lww.com/JS9/A752), pathological N0 or N2 category (Fig. S4, Supplemental Digital Content 5, http://links.lww.com/JS9/A754), or pathological T1, N3 or greater category (Fig. S5, Supplemental Digital Content 4, http://links.lww.com/JS9/A753) showed no significant correlation with systemic AC in the unadjusted subgroup analysis.

**Figure 3:**
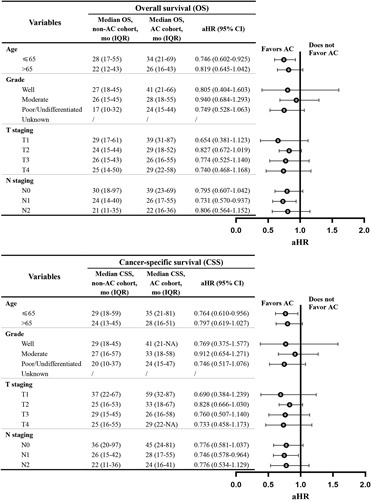
Forest plot of the association of adjuvant chemotherapy (AC) with total mortality and cancer-specific mortality in subgroup analyses.

We obtained an HR for each category in each variable with AC compared to non-AC when referring to each interaction term adjusted for age, grade, T staging, and N staging. Among these interactions, systemic AC was significantly associated with lower total mortality or cancer-specific mortality in patients younger than 65 years old and those with a pathological N1 category (Fig. 3, Table S1, Supplemental Digital Content 6, http://links.lww.com/JS9/A755).

## Discussion

Our study examined the clinical benefit and impact of systemic AC after NAC in patients with resected PDAC, using data from another national SEER database registry. Our findings further validated a significant survival benefit in PDAC patients who received AC after systemic NAC and surgical resection, compared to those who did not receive AC. After controlling for all interfering covariates, systemic AC was significantly associated with better OS and CSS in patients younger than 65 years old and those with a pathological N1 category in the matched group.

Although the use of systemic NAC has gradually increased in PDAC patients over the last decades, the indications and durations of systemic AC after NAC remain debatable. Barnes *et al*.^19^ asked in 2017: ‘Is adjuvant therapy necessary for all patients with localized pancreatic cancer who have received neoadjuvant therapy?’ and enrolled 234 consecutive PDAC patients. They discovered that, in lymph node-positive patients, the median OS with adjuvant therapy was longer than that without adjuvant therapy (29.5 vs. 23.2 months, *P*=0.02). However, no association was found in lymph node-negative patients, and it was concluded that the benefit of adjuvant therapy was limited to localized PDAC patients with positive nodes following NAC and surgery. The precise role of systemic AC was unknown, so a few retrospective studies were launched to investigate this topic^16,17,20^. van Roessel *et al*.^16^ conducted an international, multicenter, retrospective cohort study that included 520 PDAC patients receiving neoadjuvant FOLFIRINOX and resection from 31 centers in 19 countries. There was no difference in survival between patients who received AC and those who did not. After interaction analysis, AC was associated with improved OS only in patients with pathology-proven node-positive disease [26 vs. 13 months; multivariable HR, 0.41 (95% CI, 0.22–0.75); *P*=0.004]. In addition, several extensive, retrospective studies using NCDB were carried out. According to Drake *et al*.^20^, adjuvant therapy did not improve OS in PDAC patients after NAT and R0 resection, but it did improve OS in patients who underwent R1 resection. However, the Olecki *et al*. research team later reported that in their included cohort, there was a significant association between AC and improved OS (*P*<0.001). Surprisingly, the survival benefit of AC was found to be significant in patients with negative nodal disease, low-grade histology, negative margin status, and a lymph node ratio (LNR) less than 0.15, rather than in patients with high-grade histology, positive margins, and an LNR of 0.15 or higher^21^. Another NCDB-based study concluded that AC after NAC and resection was associated with improved survival, even in patients with margin-negative and node-negative disease^22^. Ma *et al*.^23^ found a survival benefit for AC compared to non-AC following NAT and surgery, especially in patients with smaller tumors (HR, 0.67, *P*<0.001 for <3.1 cm).

Given the limitations of the NCDB data, steps were taken to minimize bias. Recently, a research team from the University of Colorado School of Medicine reanalyzed NCDB data, including time period as a covariate, and found no significant difference in prognostic prediction. Even though the regimen of AC was unknown in the database, they tried their best to minimize the heterogeneity of the regimen during the specific time period. As a result, after controlling for all covariates, they found a significant association between AC and improved OS (HR=0.71; 95% CI, 0.59–0.85; *P*<0.001). AC was significantly associated with improved OS in patients younger than 75 years old, with moderately or poorly differentiated tumors, and with a pathological T category of ypT3 or greater, according to subgroup interaction analysis. Notably, the association with AC was observed regardless of pathological N category and margin status^17^. Our validation from the SEER database yielded similar results. We further validated the conclusion that there were significant associations between AC and improved OS and CSS, particularly in patients younger than 65 years old and those with a pathological N1 category after controlling for all interfering covariates. Our multivariable interaction analysis supported the previous finding that AC after NAC and surgery may benefit younger PDAC patients. However, this was the first study to incorporate the 8th edition of the AJCC N staging system into such an analysis. We only discovered that patients with N1 disease could benefit significantly from AC after NAC and surgery, rather than those with N0 or N2 diseases, which was consistent with clinical observations that the total examined and positive examined lymph nodes were significantly reduced after NAC^24–27^. According to Roland *et al*.^24^, patients with N0 disease after NAT had similar outcomes to patients with a low LNR (0.01–0.15). However, patients with an LNR greater than 0.15 had reduced survival and early recurrence, implying that patients with N2 disease may respond less to both NAT and AC. In contrast, patients with N0 disease may have already received a good response to NAT regardless of AC administration. As a result, PDAC patients with N1 disease after NAT may have a partial response to NAT, and AC may prolong the response. Another recent retrospective study from two high-volume tertiary care academic centers, conducted by Hammad *et al*.^28^, looked at adjuvant therapy’s clinical benefit and impact in PDAC patients with node-negative disease after NAT and surgical resection. They discovered a survival benefit for adjuvant therapy in patients with N0 disease after NAT and surgical resection, which may be most pronounced in patients with perineural invasion. As a result, it remained possible that systemic AC after NAT in resected PDAC patients might benefit those with invasive and metastatic potential.

Due to the limitations of the SEER database, certain vital indicators such as CA19-9, margin status, pathological response, and so on were unavailable. The proportion of patients with PDAC achieving a pathological complete response rate from NAC was nearly 3–11%, and responding patients had a significantly better prognosis due to lower rates of local recurrence, metastatic disease, and positive margins^4,29^. Macedo *et al*.^30^ also demonstrated that improved biochemical, pathological, and clinical responses associated with NAC were associated with improved OS, local recurrence-free survival (L-RFS), and metastasis-free survival (MFS), which were influenced by a significant biochemical response (CA 19-9 decrease ≥50% vs. <50%). However, according to Liu *et al*.^31^, CA19-9 normalization and decreases of more than 50% during NAC predicted no additional survival benefit from adjuvant therapy. As a result, CA19-9 variations reflecting therapeutic response and tumor biology may have an interactive effect on AC efficacy. Furthermore, as previously reported, circulating tumor cells, circulating tumor DNA, somatic tumor mutations, and biomarkers detected from treatment-naïve fine-needle biopsy specimens may be helpful predictors of NAC response, recurrence, and survival^4,32^.

## Strengths and limitations

The study’s strength is that it is a large-cohort design from a unique database that had never been used before. Concurrently, CSS and OS were calculated for survival analysis. This is the first large study to reveal associations between OS or CSS and AC after systemic NAC. However, several limitations in our study remained. First, the data in this study came from the retrospective SEER database, and it lacked critical indicators such as CA19-9, margin status, pathological response, performance status, and so on. Second, detailed regimens and durations of NAC and AC were not available here, which may have influenced response and efficacy. Third, the resection rate after NAC is unknown, and there is no data on recurrence after surgery, which may significantly impact cancer survival and treatment. As a result, a multicenter, large-scale RCT is required to eliminate all of the above limitations. A detailed study design could aid in further understanding the clinical benefit and impact of AC regimens and durations.

## Conclusions

This extensive nationwide retrospective cohort study further confirmed that AC after systemic NAC is clearly associated with improved OS and CSS in patients with resected PDAC who are younger or have aggressive tumors that may potentially respond to NAC. Further RCTs are warranted to determine whether AC is beneficial and how it affects patients, in order to develop better treatment options and recommendations.

## Ethical approval

The data used in the study are derived from a de-identified SEER database. As this study utilized secondary research involving de-identified data, it was not considered human participant research and did not require institutional review board approval.

## Consent

This study was not considered human participant research and did not require institutional review board approval because it used de-identified data from secondary research.

## Sources of funding

This study was funded by the National Natural Science Foundation of China (82103409, 82273382, 82272929, 81972218, 81972257), China Postdoctoral Science Foundation (2021M690037), Shanghai Sailing Program (21YF1407100), Shanghai ShenKang Hospital Development Centre Project (SHDC2020CR2017B), and Science and Technology Planning Project of Yunnan Province (202305AF150148). The funding agencies had no role in the study design, data collection and analyses, decision to publish, or preparation of the manuscript.

## Author contribution

W.W., S.L., and Y.X.: data acquisition, data analysis, statistical analysis, manuscript writing, and manuscript review; H.Y., Q.C., T.H., Z.X., and J.Y.: data acquisition and manuscript review; W.W.: funding acquisition, data acquisition, and manuscript review; N.P., L.L., and W.L.: funding acquisition, data acquisition, statistical analysis, manuscript writing, manuscript review, and guarantor.

## Conflicts of interest disclosure

The authors have no conflicts of interest.

## Research registration unique identifying number (UIN)

None.

## Guarantor

Ning Pu, MD, PhD, Department of Pancreatic Surgery, Zhongshan Hospital, Fudan University, 180 Fenglin Road, Shanghai 200032, People’s Republic of China, E-mail: npu15@fudan.edu.cn.

## Provenance and peer review

Not commissioned, externally peer-reviewed.

## Data availability statement

The data that support the findings of this study are openly available in the SEER database.

## Supplementary Material

SUPPLEMENTARY MATERIAL
