# Supplementary figures and images for: Survival benefit and impact of adjuvant chemotherapy following systemic neoadjuvant chemotherapy in patients with resected pancreas ductal adenocarcinoma: a retrospective cohort study
Source: Int J Surg. 2023 Jul 6;109(10):3137–46. doi: 10.1097/JS9.0000000000000589 (PMC10583928; doi:10.1097/JS9.0000000000000589)

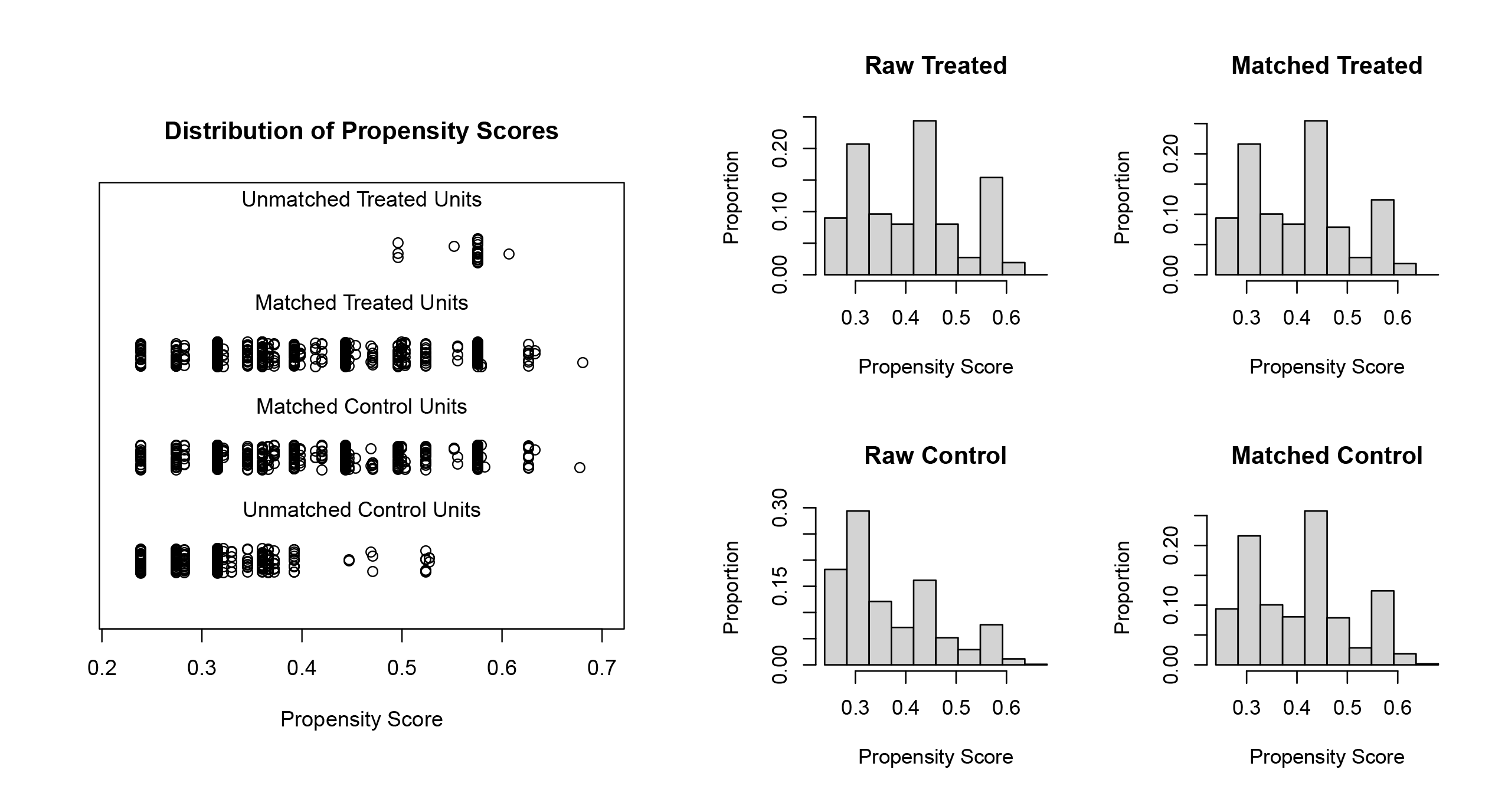

Supplement: SUPPLEMENTARY MATERIAL [file js9-109-3137-s001.tif]

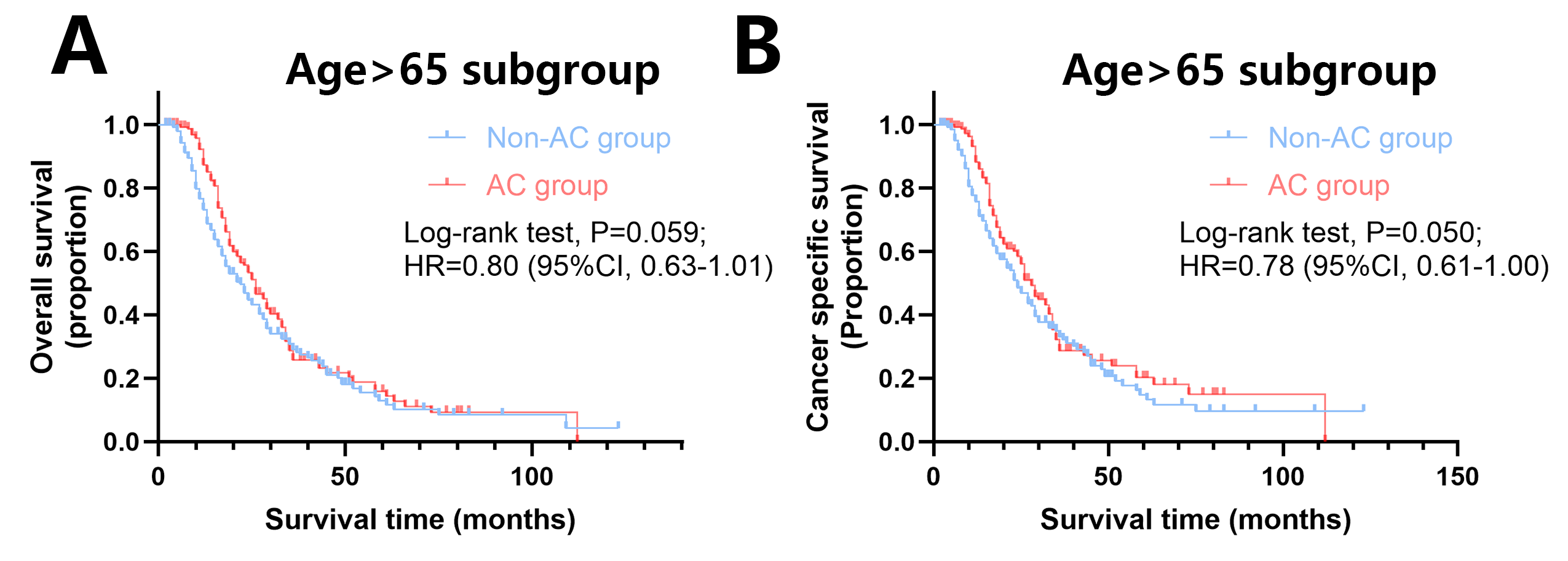

Supplement: SUPPLEMENTARY MATERIAL [file js9-109-3137-s002.tif]

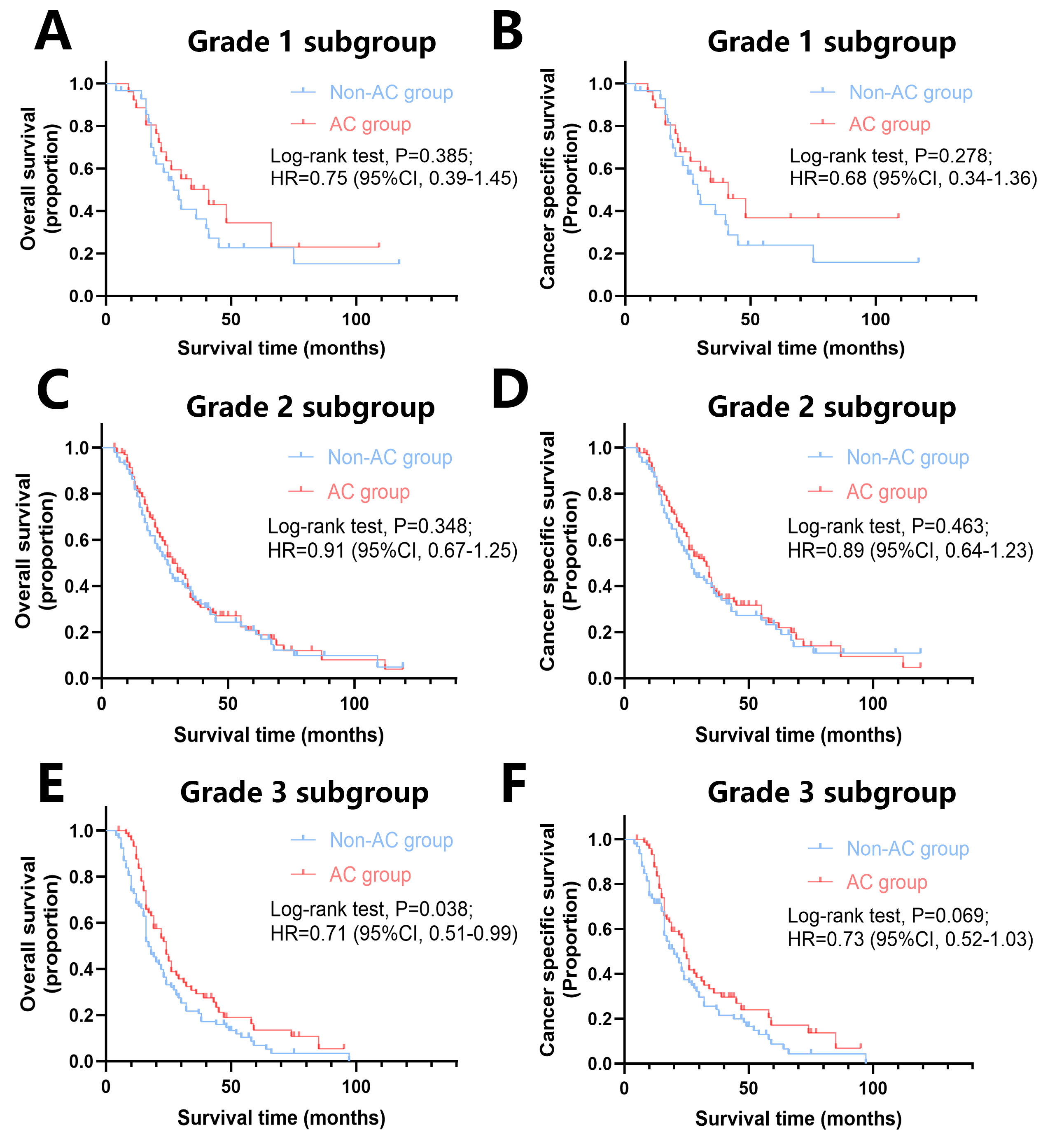

Supplement: SUPPLEMENTARY MATERIAL [file js9-109-3137-s003.tif]

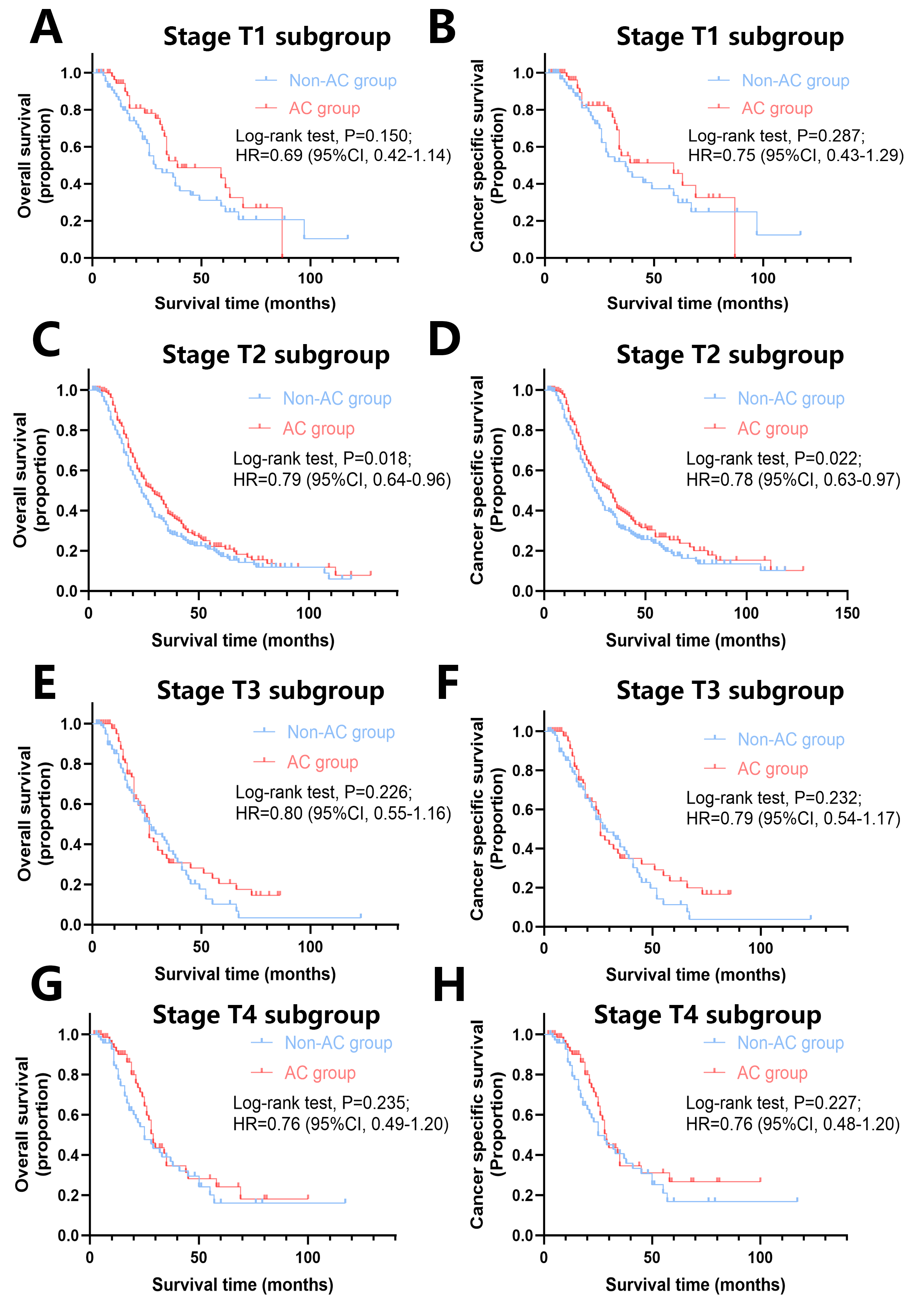

Supplement: SUPPLEMENTARY MATERIAL [file js9-109-3137-s004.tif]

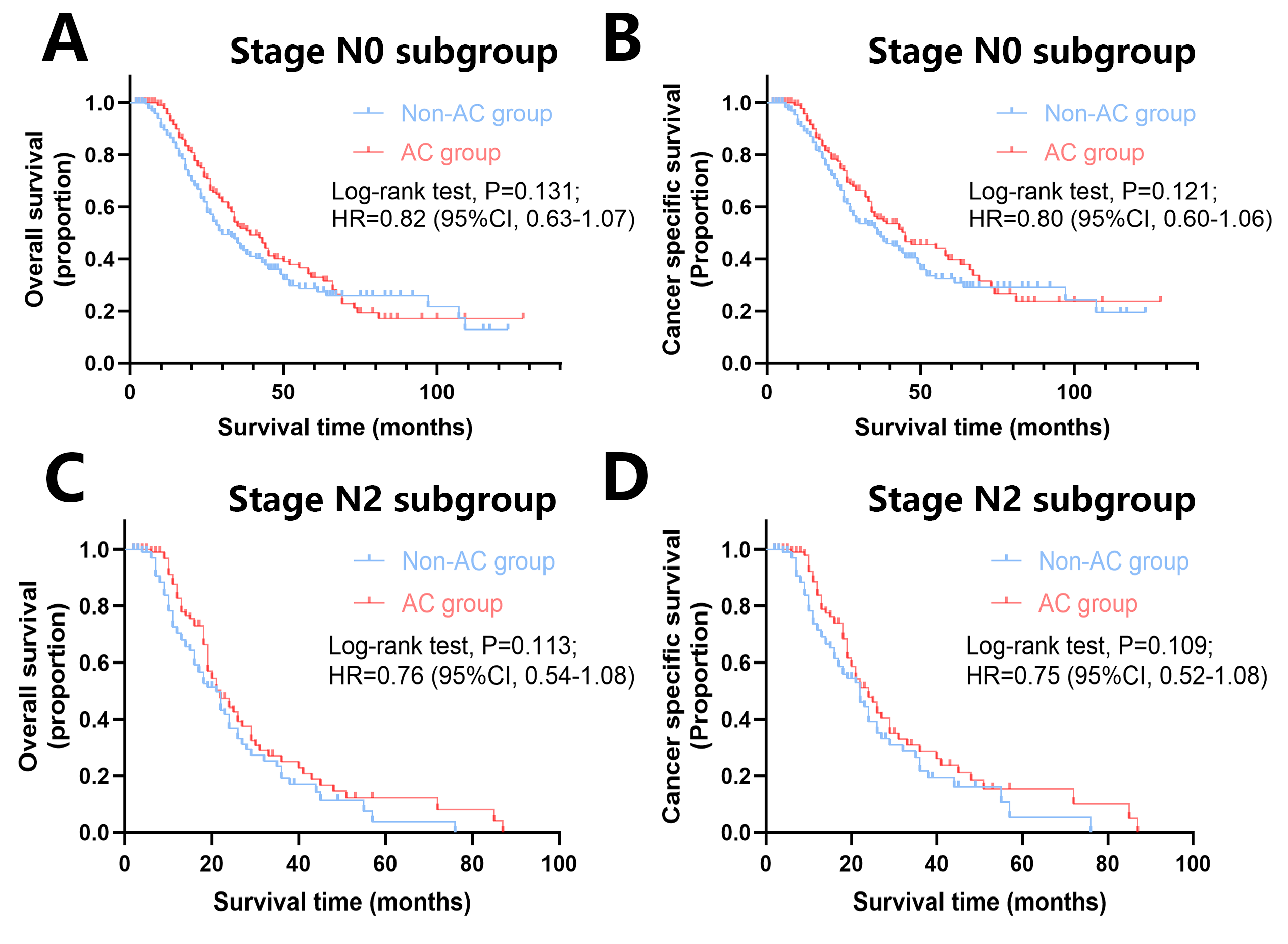

Supplement: SUPPLEMENTARY MATERIAL [file js9-109-3137-s005.tif]
