# Supplementary material for: Survival benefit and impact of adjuvant chemotherapy following systemic neoadjuvant chemotherapy in patients with resected pancreas ductal adenocarcinoma: a retrospective cohort study
Source: Int J Surg. 2023 Jul 6;109(10):3137–46. doi: 10.1097/JS9.0000000000000589 (PMC10583928; doi:10.1097/JS9.0000000000000589)
Supplement: SUPPLEMENTARY MATERIAL [file js9-109-3137-s006.docx]

**Table S1. The adjusted association of adjuvant chemotherapy (AC) with mortality in subgroup analysis.**

| **Variables** |  | | **Overall survival (OS)** | | | **Cancer-specific survival (CSS)** | | | |
| --- | --- | --- | --- | --- | --- | --- | --- | --- | --- |
|  | **Median OS, non-AC cohort, mo (IQR)** | **Median OS, AC cohort, mo (IQR)** | | **P value** | **aHR (95% CI)** | **Median CSS, non-AC cohort, mo (IQR)** | **Median CSS, AC cohort, mo (IQR)** | **P value** | **aHR (95% CI)** |
| Age  ≤65  >65 | 28 (17-55)  22 (12-43) | 34 (21-69)  26 (16-43) | | **0.008**  0.104 | 0.746 (0.602-0.925)  0.819 (0.645-1.042) | 29 (18-59)  24 (13-45) | 35 (21-81)  28 (16-51) | **0.019**  0.080 | 0.764 (0.610-0.956)  0.797 (0.619-1.027) |
| Grade  Well  Moderate  Poor/Undifferentiated  Unknown | 27 (18-45)  26 (15-45)  17 (10-32)  / | 41 (21-66)  28 (18-55)  24 (15-44)  / | | 0.537  0.705  0.106  / | 0.805 (0.404-1.603)  0.940 (0.684-1.293)  0.749 (0.528-1.063)  / | 29 (18-45)  27 (16-57)  20 (10-37)  / | 41 (21-NA)  33 (18-58)  24 (15-47)  / | 0.473  0.585  0.117  / | 0.769 (0.375-1.577)  0.912 (0.654-1.271)  0.746 (0.517-1.076)  / |
| T classification  T1  T2  T3  T4 | 29 (17-61)  24 (15-44)  26 (15-43)  25 (14-50) | 39 (31-87)  29 (18-52)  26 (16-55)  29 (22-58) | | 0.214  0.074  0.195  0.196 | 0.654 (0.381-1.123)  0.827 (0.672-1.019)  0.774 (0.525-1.140)  0.740 (0.468-1.168) | 37 (22-67)  25 (16-53)  29 (15-45)  25 (16-55) | 59 (32-87)  33 (18-67)  26 (16-58)  29 (22-NA) | 0.214  0.090  0.185  0.196 | 0.690 (0.384-1.239)  0.828 (0.666-1.030)  0.760 (0.507-1.140)  0.733 (0.458-1.173) |
| N classification  N0  N1  N2 | 30 (18-97)  24 (14-40)  21 (11-35) | 39 (23-69)  26 (17-55)  22 (16-36) | | 0.097  **0.013**  0.237 | 0.795 (0.607-1.042)  0.731 (0.570-0.937)  0.806 (0.564-1.152) | 36 (20-97)  26 (15-42)  22 (11-36) | 45 (24-81)  28 (17-55)  24 (16-41) | 0.087  **0.025**  0.185 | 0.776 (0.581-1.037)  0.746 (0.578-0.964)  0.776 (0.534-1.129) |
